# Eosinophilic esophagitis is associated with increased risk of allergic conjunctivitis: a multicenter cohort study

**DOI:** 10.1186/s12348-026-00607-9

**Published:** 2026-06-03

**Authors:** Natan Lishinsky-Fischer, Daniel Burg, Shoham Kubovsky, Yossi Eshel, Jaime Levy

**Affiliations:** 1 Department of Ophthalmology, Hadassah Medical Center, Faculty of Medicine, Hebrew University of Jerusalem, Kiryat Hadassah, POB 12000, Jerusalem, 91120 Israel; 2 Faculty of Medicine, The Hebrew University of Jerusalem, Jerusalem, Israel

**Keywords:** Eosinophilic esophagitis, Ocular comorbidities, Allergic conjunctivitis, Atopy, Electronic health records

## Abstract

**Background:**

Eosinophilic esophagitis (EoE) is a chronic, Th2-driven inflammatory esophageal disease frequently associated with atopic disorders. However, ocular comorbidities in EoE, particularly allergic conjunctivitis, have not been well characterized. In this study, we compare the incidence of ocular outcomes, with a focus on allergic conjunctivitis, between patients with EoE and matched controls with gastroesophageal reflux disease with esophagitis (GERD-E).

**Methods:**

We conducted a retrospective, propensity score-matched cohort study using the TriNetX Global Collaborative Network, including 23,488 patients with EoE and 23,488 matched GERD-E controls. Eligible patients had at least one ophthalmology encounter ≥ 30 days after the index diagnosis. The primary outcome was allergic conjunctivitis; secondary outcomes included dry eye syndrome, keratoconjunctivitis sicca, blepharitis, meibomian gland dysfunction, and uveitis. Kaplan-Meier analyses, log-rank tests, and Cox proportional hazards models were used to estimate hazard ratios (HRs) over a 3-year follow-up period.

**Results:**

EoE was associated with a higher 3-year risk of allergic conjunctivitis compared with GERD-E (HR 1.42; 95% CI [1.20,– 1.69]; *p* < 0.001). No significant differences were observed for dry eye syndrome, keratoconjunctivitis sicca, blepharitis, meibomian gland dysfunction, or uveitis. Positive and negative control outcomes supported internal validity.

**Conclusions:**

Patients with EoE have an elevated risk of allergic conjunctivitis compared with GERD-E, consistent with the systemic Th2 inflammatory profile of EoE. Other ocular comorbidities were not significantly increased. These findings highlight the importance of multidisciplinary, allergy-aware care in patients with EoE.

## Introduction

Eosinophilic esophagitis (EoE) is a chronic, immune-mediated esophageal disease characterized by symptoms of esophageal dysfunction and histologic eosinophilic infiltration in the absence of competing etiologies [12]. Clinically and biologically, EoE is part of the T helper type 2 (Th2) spectrum of allergic diseases [1–3]. Patients frequently exhibit comorbid atopic conditions such as allergic rhinitis, asthma, and atopic dermatitis, consistent with the concept of “allergic march”, which spans multiple mucosal surfaces and barrier organs [4–6]. In contrast, gastroesophageal reflux disease (GERD) is primarily driven by mechanical and chemical injury from gastric refluxate, often exacerbated by anatomic abnormalities such as diaphragmatic or hiatal hernias [7, 8].

Ocular allergic disease is both common and clinically relevant. In population-based samples, allergic conjunctivitis is typically reported in about 6% to 30% of individuals, although estimates vary with season, region, age structure, and diagnostic criteria. Among people with atopic disease, a pooled prevalence of approximately 31.7% has been reported, with a 95% confidence interval of about 27.7% to 35.9% [9, 10]. These gradients mirror the systemic nature of type-2 inflammation and raise a biologically plausible association between EoE and ocular allergic complications. While EoE’s atopic profile is well known, few studies have used real-world, multicenter data to assess ocular outcomes specifically in EoE compared to GERD-E.

In this study, we assessed the association between EoE and ocular outcomes using a large, multi-institutional dataset derived from routine clinical care.

## Methods

### Study design and data source

This retrospective cohort study was conducted using the TriNetX research network, which provides standardized access to demographic information, diagnoses, procedures, medications, laboratory results, and healthcare utilization metrics. Participating sites are federated into several networks that are typically organized by geographic regions. For the current analysis, we utilized the Global Collaborative Network, which collects records from health care organizations across multiple countries [11]. Because TriNetX functions as a dynamic resource, the underlying datasets are refreshed regularly, with new information contributed on a near-daily basis. As of October 14th, 2025, the Global Collaborative Network contained electronic healthcare records (EHRs) of 195,161,097 patients from 162 healthcare organizations (HCOs) worldwide. In line with TriNetX privacy policies, the names and locations of participating health care organizations are not disclosed. The platform operates in compliance with the Health Insurance Portability and Accountability Act (HIPAA). Because only deidentified data were accessed for this analysis, institutional review board review was not required and the study qualified for exemption.

Study variables were coded using the International Classification of Diseases, Tenth Revision, Clinical Modification (ICD-10-CM). Medication exposures were identified via RxNorm and the Anatomical Therapeutic Chemical (ATC) classification. The analysis was executed with the platform’s Compare Outcomes workflow and time-stamped October 14th, 2025. Data included deidentified diagnoses, procedures, and medication records from participating HCOs.

### Cohort definitions

We identified two mutually exclusive cohorts: an EoE cohort: ICD-10-CM K20.0 and a GERD-E cohort: ICD-10-CM K21.0. Patients with other major gastrointestinal diseases or malignancies were excluded, including eosinophilic gastritis or gastroenteritis (K52.81), eosinophilic colitis (K52.82), hypereosinophilic syndromes (D72.11), Crohn’s disease (K50), ulcerative colitis (K51), celiac disease (K90.0), malignant neoplasm of esophagus (C15), Barrett’s esophagus (K22.7). Cohorts were mutually exclusive; individuals with diagnostic codes for both EoE and GERD-E were excluded from analysis. To ensure consistent outcome capture, eligibility required at least one ophthalmology encounter occurring at least 30 days after the index date (CPT 92002, 92004, 92012, 92014, 92285, 92286, 92015, 99213, 99214).

### Index event and follow-up

The index event was the first qualifying diagnosis of EoE or GERD-E. Outcomes were assessed from 30 days to 3 years post-index. Individuals with the outcome recorded before this window were excluded.

### Propensity score-matching

To minimize baseline imbalances, one-to-one propensity score matching (PSM) was performed using the TriNetX built-in matching algorithm with a caliper of 0.1 pooled standard deviations. Balance between cohorts was assessed using standardized mean differences (SMD), with values < 0.1 indicating adequate balance.

Demographic variables were used including age at index, current age, race/ethnicity, sex, and, including categories for Black or African American (OMB code 2054-5), White (2106-3), Asian (2028-9), Hispanic or Latino (2135-2). Clinical comorbidities were identified based on ICD-10-CM, RxNorm and CPT coding systems and included diabetes mellitus (E08-E13), hypertensive diseases (I10-I1A), Disorders of lipoprotein metabolism and other lipidemias (E78), Overweight and obesity (E66), Vasomotor and allergic rhinitis (J30), Asthma (J45), Atopic dermatitis (L20), Nasal polyp (J33), Blepharitis (H01.0), Meibomian gland dysfunction of eyelid (H02.88), Dry eye syndrome (H04.12), Keratoconjunctivitis sicca, not specified as Sjögren’s (H16.22), Acute atopic conjunctivitis (H10.1), Vernal conjunctivitis (H10.44), Sjögren syndrome (M35.0), Neurotrophic keratoconjunctivitis (H16.23), Retinal vascular occlusions (H34), Retinal vasculitis (H35.06), Retinal telangiectasis (H35.07), Chorioretinal inflammation (H30), Iridocyclitis (H20), Panuveitis (H44.11). Medication exposures included administration of omeprazole (RxNorm 7646), pantoprazole (RxNorm 40790), famotidine (RxNorm 4278), Glucocorticoids (ATC H02AB), lifitegrast (RxNorm 1801820), olopatadine (RxNorm 135391), ketotifen (RxNorm 6146).

### Outcomes

The primary ocular outcome was allergic conjunctivitis (ICD-10-CM H10.1, H10.44, H10.45). Secondary outcomes included dry eye syndrome (H04.12), keratoconjunctivitis sicca not specified as Sjögren’s (H16.22), blepharitis (H01.0), meibomian gland dysfunction of eyelid (H02.88), and uveitis, defined by iridocyclitis (H20), chorioretinal inflammation (H30), panuveitis (H44.11), retinal vasculitis (H35.06), or sympathetic uveitis (H44.13).

In addition to the primary outcomes, we prespecified positive and negative control outcomes to assess internal validity. Diaphragmatic hernia (K44) was selected as a positive control outcome due to its established association with reflux phenotypes, whereas breast cancer (C50) served as a negative control with no expected relationship to the exposures of interest. Both control outcomes were evaluated over a follow-up period of 30 days to 3 years after the index event. For each outcome, patients who had a recorded history of that condition prior to the start of the follow-up period were excluded from the analysis.

### Statistical Analysis

We generated Kaplan-Meier curves within the TriNetX “Compare Outcomes” module. We applied the censoring criteria described and conducted separate analyses for each outcome, including log-rank tests and Cox proportional-hazards models to estimate hazard ratios (HRs) with 95% confidence intervals. Following the analysis, we reported (1) the total number of patients in each cohort after excluding individuals with pre-existing evidence of the outcome prior to the follow-up window; and (2) the number of patients who experienced the outcome during follow-up. (3) For each outcome, the cumulative incidence at the end of follow-up was calculated automatically by the platform and expressed as the proportion of patients experiencing the event among those at risk. (4) Survival probability throughout the follow-up period was estimated using the Kaplan-Meier product-limit method. When two cohorts were compared, a log-rank test was used to determine whether the survival curves differed, based on the chi-square (χ2) statistic. Cox proportional hazards models were then fitted to estimate the hazard ratio (HR) together with its 95% confidence interval. HR represents the ratio of the instantaneous risk of experiencing the outcome for the first time between the two cohorts. Lastly, the p-value for the proportional hazards assumption is reported, testing whether the hazard functions for the cohorts are proportional over time-that is, whether they follow the same time pattern and differ only by a constant multiplicative factor. Both HR estimates and the proportionality values are computed within the TriNetX platform, which runs a suite of tests using R’s Survival package (version 3.2-3) and comparing the numbers with output from SAS (version 9.4) in order to validate the results. Statistical significance was defined as a log-rank test *p* < 0.05 and a proportionality test *p* > 0.01.

## Results

### Population Characteristics

Before PSM, 23,540 individuals met the case-definition criteria for EoE and 667,343 for GERD-E. Patients in the GERD-E cohort were older on average (mean age 54.1 vs. 28.9 years) and included a lower proportion of males (36.6% vs. 62.3%). Racial and ethnic distributions also differed: the GERD-E cohort had a slightly higher proportion of Hispanic (8.1% vs. 5.6%) and Black patients (11.3% vs. 6.3%). Comorbidities, including hypertension, diabetes, nicotine dependence, disorders of lipoprotein metabolism and other lipidemias, overweight and obesity, were more prevalent in the GERD-E cohort. In contrast, EoE showed a higher prevalence of features associated with the “atopic march”, including asthma, atopic dermatitis, acute atopic conjunctivitis and vasomotor and allergic rhinitis. Medication exposures (omeprazole, pantoprazole, famotidine, glucocorticoids, lifitegrast, olopatadine), were higher among GERD-E patients. In contrast with EoE, in which the use of ketotifen and olopatadine was slightly higher. After PSM, the matched cohorts each included 23,488 patients and were well-balanced across most clinical, demographic, and medication variables. Standardized mean differences for sex, age, race, comorbidities and medication use services were substantially reduced, generally below 0.05. Table 1 presents the characteristics of both cohorts, before and after PSM.

**Table 1:** Baseline characteristics before and after propensity score matching (PSM) for patients with eosinophilic esophagitis (EoE) and patients with gastroesophageal reflux disease with esophagitis (GERD-E) over 3 years in the Global Collaborative Network. Abbreviations: SMD, standardized mean difference. Bold text indicates covariates with residual imbalance after matching (SMD > 0.1 and *P* < 0.05) between the two cohorts

Characteristic Name	Before PSM				After PSM			
	EoE (*n* = 23,540)	GERD-E (*n* = 667,343)	*P*	Std diff.	EoE (*n* = 23,488)	GERD-E (*n* = 23,488)	*P*	Std diff.
Age at Index (mean ± SD)	28.85 ± 20.05	54.11 ± 20.23	**< 0.0001**	**1.25**	28.9 ± 20.04	29.29 ± 20.31	0.0389	0.02
White (%)	18,518 (78.67)	468,995 (71.53)	**< 0.0001**	**0.17**	18,473 (78.65)	18,680 (79.53)	0.0188	0.02
Black or African American (%)	1493 (6.34)	74,402 (11.35)	**< 0.0001**	**0.18**	1493 (6.36)	1375 (5.85)	0.0230	0.02
Asian (%)	451 (1.92)	20,156 (3.07)	< 0.0001	0.07	451 (1.92)	389 (1.66)	0.0309	0.02
Hispanic or Latino (%)	1307 (5.55)	53,006 (8.08)	**< 0.0001**	**0.10**	1307 (5.56)	1209 (5.15)	0.0446	0.02
Male (%)	14,662 (62.28)	240,154 (36.63)	**< 0.0001**	**0.53**	14,610 (62.2)	14,564 (62.01)	0.6618	0.00
Female (%)	8345 (35.45)	376,806 (57.47)	**< 0.0001**	**0.45**	8345 (35.53)	8405 (35.78)	0.5633	0.01
Hypertensive diseases (%)	2616 (11.11)	333,005 (50.79)	**< 0.0001**	**0.95**	2616 (11.14)	2591 (11.03)	0.7133	0.00
Diabetes mellitus (%)	859 (3.65)	137,521 (20.97)	**< 0.0001**	**0.55**	859 (3.66)	809 (3.44)	0.2125	0.01
Disorders of lipoprotein metabolism and other lipidemias (%)	2936 (12.47)	320,780 (48.92)	**< 0.0001**	**0.86**	2936 (12.5)	2940 (12.52)	0.9555	0.00
Overweight and obesity (%)	2082 (8.85)	177,569 (27.08)	**< 0.0001**	**0.49**	2082 (8.86)	2067 (8.8)	0.8073	0.00
Nicotine dependence (%)	616 (2.62)	87,315 (13.32)	**< 0.0001**	**0.40**	616 (2.62)	576 (2.45)	0.2406	0.01
Asthma (%)	4618 (19.62)	103,790 (15.83)	**< 0.0001**	**0.10**	4571 (19.46)	4606 (19.61)	0.6838	0.00
Vasomotor and allergic rhinitis (%)	4431 (18.82)	101,988 (15.55)	< 0.0001	0.09	4388 (18.68)	4343 (18.49)	0.5935	0.00
Atopic dermatitis (%)	1229 (5.22)	9216 (1.41)	**< 0.0001**	**0.21**	1182 (5.03)	977 (4.16)	< 0.0001	0.04
Nasal polyp (%)	105 (0.45)	2696 (0.41)	0.4120	0.01	104 (0.44)	66 (0.28)	0.0035	0.03
Dry eye syndrome (%)	230 (0.98)	27,373 (4.18)	**< 0.0001**	**0.20**	230 (0.98)	155 (0.66)	0.0001	0.04
Keratoconjunctivitis sicca, not specified as Sjogren’s (%)	32 (0.14)	3286 (0.5)	< 0.0001	0.06	32 (0.14)	21 (0.09)	0.1306	0.01
Blepharitis (%)	78 (0.33)	7990 (1.22)	**< 0.0001**	**0.10**	78 (0.33)	63 (0.27)	0.2058	0.01
Meibomian gland dysfunction of eyelid (%)	59 (0.25)	4143 (0.63)	< 0.0001	0.06	59 (0.25)	50 (0.21)	0.3881	0.01
Presence of spectacles and contact lenses (%)	47 (0.2)	2102 (0.32)	0.0012	0.02	47 (0.2)	30 (0.13)	0.0525	0.02
Acute atopic conjunctivitis (%)	486 (2.06)	6517 (0.99)	< 0.0001	0.09	464 (1.98)	397 (1.69)	0.0212	0.02
Vernal conjunctivitis (%)	10 (0.04)	35 (0.0)	< 0.0001	0.02	10 (0.04)	10 (0.04)	1.0000	0.00
Olopatadine (%)	289 (1.23)	7355 (1.12)	0.1299	0.01	286 (1.22)	237 (1.01)	0.0312	0.02
Ketotifen (%)	123 (0.52)	2534 (0.39)	0.0010	0.02	121 (0.52)	105 (0.45)	0.2860	0.01
Lifitegrast (%)	15 (0.06)	1066 (0.16)	0.0002	0.03	15 (0.06)	10 (0.04)	0.3172	0.01
Iridocyclitis (%)	36 (0.15)	2214 (0.34)	< 0.0001	0.04	36 (0.15)	28 (0.12)	0.3170	0.01
Panuveitis (%)	10 (0.04)	122 (0.02)	0.0098	0.01	10 (0.04)	10 (0.04)	1.0000	0.00
Chorioretinal inflammation (%)	10 (0.04)	450 (0.07)	0.1297	0.01	10 (0.04)	11 (0.05)	0.8272	0.00
Neurotrophic keratoconjunctivitis (%)	10 (0.04)	104 (0.02)	0.0020	0.02	10 (0.04)	10 (0.04)	1.0000	0.00
Retinal vascular occlusions (%)	22 (0.09)	2397 (0.37)	< 0.0001	0.06	22 (0.09)	26 (0.11)	0.5635	0.01
Retinal vasculitis (%)	10 (0.04)	90 (0.01)	0.0004	0.02	10 (0.04)	10 (0.04)	1.0000	0.00
Retinal telangiectasis (%)	10 (0.04)	100 (0.02)	0.0013	0.02	10 (0.04)	0 (0.0)	0.0016	0.03
Glucocorticoids (%)	8065 (34.26)	325,185 (49.59)	**< 0.0001**	**0.31**	8032 (34.2)	8066 (34.34)	0.7410	0.00
Omeprazole (%)	7033 (29.88)	223,519 (34.09)	< 0.0001	0.09	7021 (29.89)	7144 (30.42)	0.2162	0.01
Pantoprazole (%)	3243 (13.78)	216,078 (32.95)	**< 0.0001**	**0.47**	3242 (13.8)	3304 (14.07)	0.4088	0.01
Famotidine (%)	1860 (7.9)	148,262 (22.61)	**< 0.0001**	**0.42**	1860 (7.92)	1708 (7.27)	0.0081	0.02
Sjogren’s syndrome (%)	85 (0.36)	6589 (1.0)	< 0.0001	0.08	85 (0.36)	64 (0.27)	0.0849	0.02

### Positive and negative controls

During the 3-year follow-up, patients with EoE showed a markedly lower risk of diaphragmatic hernia, compared to patients with GERD-E (HR 0.550; 95% CI [0.470, 0.642]; *p* < 0.0001). In contrast, breast cancer was not significantly different between the groups with HR of 0.8 (95% CI [0.48, 1.34]; *p* = 0.3913).

### Main outcomes

**Fig. 1:**
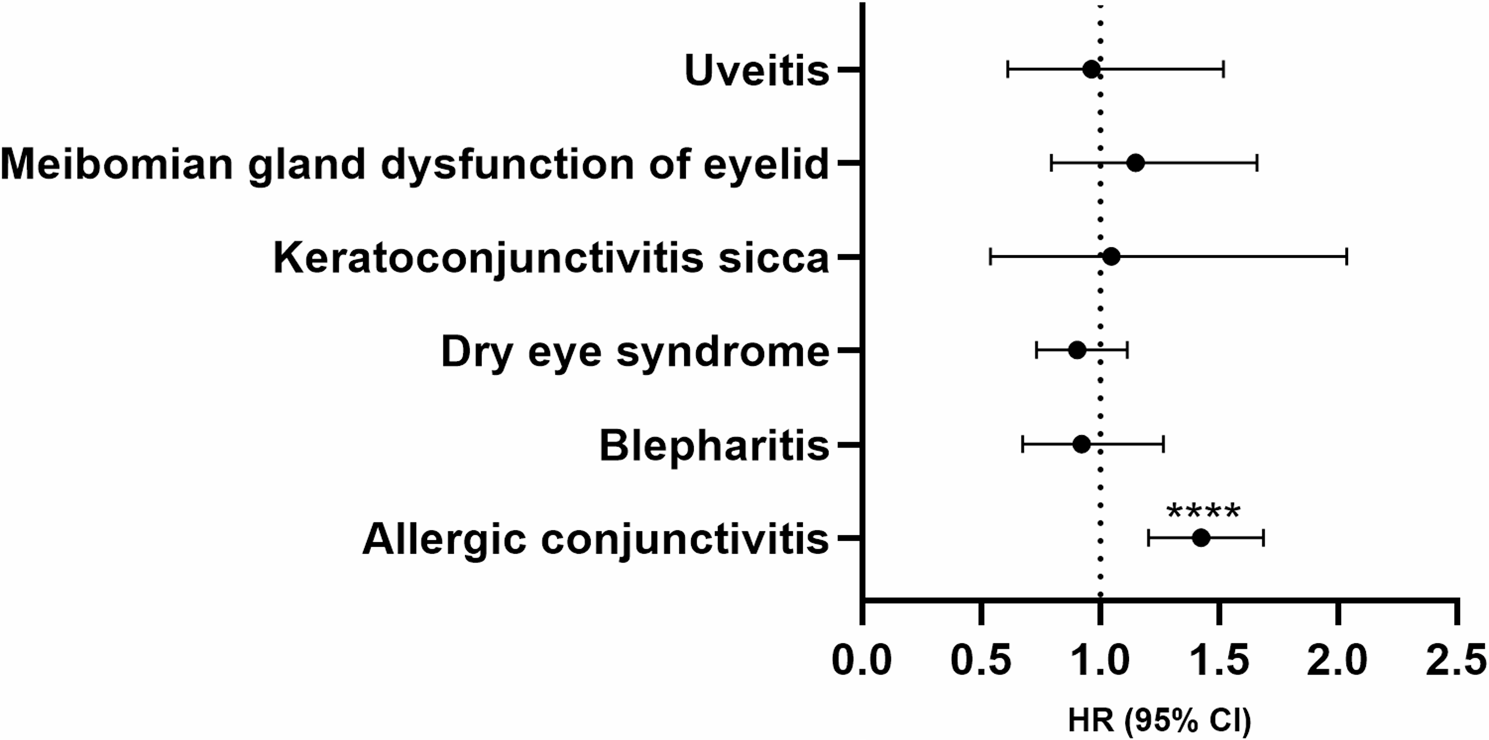
Hazard ratios for ocular and systemic outcomes in eosinophilic esophagitis vs. GERD With Esophagitis. Forest plot of hazard ratios (HRs) with 95% CIs for ocular outcomes in eosinophilic esophagitis (EoE) vs. gastroesophageal reflux disease with esophagitis (GERD-E) after 1:1 propensity score matching. Outcomes shown (top to bottom): uveitis, meibomian gland dysfunction of eyelid, keratoconjunctivitis sicca, dry eye syndrome, blepharitis, and allergic conjunctivitis. Outcomes were evaluated from 30-days to 3-years after the index diagnosis in the TriNetX Global Collaborative Network. **** denotes *p* < 0.0001 (log-rank test)

During the 3-year follow-up, patients with EoE had a significantly higher risk of allergic conjunctivitis compared with the GERD-E cohort, with HR of 1.424 (95% CI [1.202, 1.687]; *p* < 0.0001). Other outcomes showed no significant associations with EoE. Specifically, dry eye syndrome (HR = 0.903; 95% CI [0.733, 1.114]; *p* = 0.342), blepharitis (HR = 0.923; 95% CI [0.673, 1.265]; *p* = 0.617), meibomian Gland dysfunction of the eyelid (HR = 1.148; 95% CI [0.795, 1.659]; *p* = 0.462), uveitis (HR = 0.964; 95% CI [0.612, 1.518]; *p* = 0.176), and keratoconjunctivitis sicca (HR = 1.046; 95% CI [0.538, 2.037]; *p* = 0.894) were not significantly different between groups. These results are illustrated and summarized in Fig. 1; Table 2.

**Table 2:** Summary of hazard ratios (HRs) with 95% confidence intervals (CIs), log-rank P values, and proportional-hazards assumption p-values for allergic conjunctivitis, dry eye syndrome, keratoconjunctivitis sicca, blepharitis, meibomian gland dysfunction, uveitis, diaphragmatic hernia, and breast cancer in patients with eosinophilic esophagitis (EoE) and patients with gastroesophageal reflux disease with esophagitis (GERD-E), after 1:1 propensity score matching

Outcome	Patients in cohort		Patients with outcome		Incident risk (%)		Survival probability at the end of time window		HR [95% CI]	Log-rank test *p*-value	Proportionality test *p*-value
	EoE	GERD-E	EoE	GERD-E	EoE	GERD-E	EoE	GERD-E			
**Allergic conjunctivitis**	**22,583**	**22,702**	**289**	**251**	**1.2797**	**1.1056**	**0.9797**	**0.9856**	**1.42 [1.2, 1.69]**	**< 0.0001**	**0.8407**
Dry eye syndrome	23,098	23,138	151	209	0.6537	0.9033	0.9893	0.9880	0.9 [0.73, 1.11]	0.342	0.8544
Keratoconjunctivitis sicca	23,433	23,450	16	19	0.0683	0.0810	0.9989	0.9989	1.05 [0.54, 2.04]	0.8939	0.555
Blepharitis	23,307	23,294	67	92	0.2875	0.3950	0.9950	0.9946	0.92 [0.67, 1.26]	0.6169	0.3378
Meibomian gland dysfunction of eyelid	23,398	23,410	54	60	0.2308	0.2563	0.9961	0.9965	1.15 [0.79, 1.66]	0.4616	0.3947
Uveitis	23,375	23,385	33	43	0.1412	0.1839	0.9977	0.9975	0.96 [0.61, 1.52]	0.8743	0.1759
**Diaphragmatic hernia**	**22,058**	**20,853**	**235**	**490**	**1.0654**	**2.3498**	**0.9832**	**0.9701**	**0.55 [0.47**,** 0.64]**	**< 0.0001**	**0.1837**
Breast cancer	23,357	23,372	24	37	0.1028	0.1583	0.9983	0.9980	0.8 [0.48, 1.34]	0.3913	0.4814

## Discussion

In this large, propensity-matched, multi-institutional cohort, patients with EoE had a significantly higher 3-year risk of allergic conjunctivitis compared with GERD-E, while risks for other ocular outcomes (dry eye syndrome, keratoconjunctivitis sicca, blepharitis, meibomian gland dysfunction, and uveitis) did not differ.

The conjunctivitis signal coheres with the atopy-enriched profile of EoE and the broader “allergic march.” Type-2 cytokines (e.g., IL-4/IL-13/IL-5), IgE-mediated mast-cell activation, and epithelial barrier dysfunction provide shared mechanistic threads across airway, skin, and ocular mucosa [12–21]. Epidemiologic range of ~ 6 to 30% allergic conjunctivitis prevalence in the general population, rising to 31.7% (95% CI [27.7,35.9]) in atopic populations lend weight to the expectation that an atopic, Th2-skewed condition such as EoE would carry elevated ocular-allergy risk relative to GERD-E [9, 10]. While causality cannot be inferred from our observational design, the direction and magnitude of association are clinically sensible given these gradients. From an ophthalmic perspective, allergic conjunctivitis represents a prototypical form of ocular surface inflammation, characterized by epithelial barrier dysfunction, mast-cell activation, and type-2 cytokine signaling at the conjunctival mucosa. The observed association between EoE and allergic conjunctivitis therefore supports the concept that systemic Th2-driven disease can manifest as clinically relevant inflammatory pathology at the ocular surface.

Prior literature consistently demonstrates the high atopic burden accompanying EoE across the life course [4]. In children, the most prevalent comorbidity is rhinoconjunctivitis, affecting 60% of children with EoE; reported rates of asthma and atopic dermatitis are 59.8% and 17.8% in the pediatric population, respectively [22, 23]. A meta-analysis quantified this enrichment: the odds of allergic rhinitis in EoE are ~ 5-fold higher than in non-EoE populations (odds ratio [OR] 5.09, 95% CI [2.91, 8.90]), and the odds of asthma are ~ 3-fold higher (OR 3.01, 95% CI [1.96, 4.62]) [22, 23]. With respect to ocular allergy specifically, allergic conjunctivitis occurs in approximately 60% of the pediatric population with EoE; in adults with EoE, rhinoconjunctivitis is likewise the most common allergic diagnosis, mirroring pediatric patterns [4, 22]. Notably, one prospective cohort reported universal allergic multimorbidity among EoE patients (i.e., every participant had ≥ 1 additional allergic disease beyond EoE) [24].

Despite this robust atopic signal, the field has lacked large, multicenter, epidemiologic comparisons of ocular outcomes between EoE and GERD. Most prior studies have profiled overall atopy rather than specific ocular endpoints such as allergic conjunctivitis, leaving a gap our analysis was designed to address [4, 22, 23, 25, 26]. In that context, our findings extend the atopic profile of EoE to the ocular surface in a matched, real-world comparison against GERD with esophagitis and provide a quantitative risk estimate for allergic conjunctivitis. Conversely, our replication of the diaphragmatic hernia signal in GERD strengthens internal consistency and supports the biological specificity of the associations we observed.

### Clinical implications

For EoE, our data support incorporating brief ocular allergy screening into routine clinical visits, including questions about itching, tearing, redness, and photophobia. Early identification of ocular involvement may help prevent chronic irritation and improve patient quality of life. Clinicians should maintain a low threshold for referral to ophthalmology or allergy specialists, particularly in patients with persistent or recurrent symptoms. In addition, awareness of the potential overlap between EoE and ocular surface disorders may facilitate more comprehensive multidisciplinary care, enhance symptom control, and contribute to better long-term outcomes.

### Methodological considerations and limitations

Several methodological aspects and potential sources of bias were considered prior to conducting this analysis: (1) ICD-10 ascertainment and algorithm choice. Cohorts were defined using ICD-10 K20.0 for EoE and K21.0 for GERD with esophagitis, with mutually exclusive exclusions. Validation studies quantify K20.0 performance: in pediatrics, Robson et al. reported specificity 99% and sensitivity 61% for identifying true EoE [27]. In adults within the U.S. Veterans Affairs system, Low et al. showed that stricter claims/EHR algorithms-e.g., ≥ 2 encounters with K20.0 separated by > 30 days-achieve PPV 93.3%, while sensitivity remains limited (37 to 56%) and specificity ~ 99% [28]. These metrics imply that K20.0-based cohorts are highly specific but under-ascertain true EoE, which would bias effect estimates toward the null (i.e., conservative HRs rather than exaggerated differences). (2) Selection by care utilization. Requiring an ophthalmology encounter ≥ 30 days post-index standardizes opportunity for outcome detection but enriches for patients who access eye care, potentially inflating absolute event capture (applied symmetrically to both arms). (3) Temporal design. Follow-up began 30 days after index and continued to 1095 days; differential observed follow-up is addressed by Kaplan-Meier methods but may still interact with utilization patterns. (4) Residual confounding and phenotype granularity. ICD-10 codes may not fully resolve ocular allergy subtypes; environmental exposures and therapy intensity were not modeled. (5) Generalizability. TriNetX site mix may under-represent certain populations/geographies; however, multi-site breadth and standardized analytics mitigate single-center biases.

## Future directions

Prospective studies with standardized ocular phenotyping and biomarker profiling (e.g., tear cytokines, serum IgE, eosinophil activity markers) could clarify mechanisms linking esophageal type-2 inflammation to ocular allergy. Analyses stratified by EoE therapy class (proton-pump inhibitors, swallowed topical steroids, elimination diets, biologics) may reveal whether anti-type-2 treatments modify ocular risk. Expanding beyond K21.0 to include GERD without esophagitis would test generalizability across the broader GERD spectrum.

## Conclusions

Compared with GERD-E, EoE is associated with a higher 3-year risk of allergic conjunctivitis in real-world practice, consistent with its atopic biology. These findings support multidisciplinary, allergy-aware care for EoE and anatomy-informed management for GERD-E.

## Data Availability

The datasets generated during and/or analyzed during the current study are available from the corresponding author on reasonable request.

## Abbreviations

EoE: Eosinophilic esophagitis:
GERD-E: Gastroesophageal reflux disease with esophagitis
HRs: Hazard ratios
Th2: T helper type 2
EHRs: electronic healthcare records
HCOs: healthcare organizations
HIPAA: Health Insurance Portability and Accountability Act
ICD-10-CM: International Classification of Diseases, Tenth Revision, Clinical Modification
ATC: Anatomical Therapeutic Chemical classification
PSM: Propensity score matching

## References

[1] Muir A, Falk GW (2021) Eosinophilic Esophagitis JAMA 326:1310 34609446 10.1001/jama.2021.14920 PMC9045493

[2] Dellon ES et al (2025) ACG Clinical Guideline: Diagnosis and Management of Eosinophilic Esophagitis. Am J Gastroenterol 120:31–59 39745304 10.14309/ajg.0000000000003194

[3] Aziz S et al (2025) Unravelling the pathogenesis of eosinophilic esophagitis from genetic predisposition to environmental triggers. Clin Exp Immunol 219 10.1093/cei/uxaf039 PMC12232548 40581073

[4] Domenech Witek J et al (2023) Description of allergic phenotype in patients with eosinophilic oesophagitis: management protocol proposal. Sci Rep 13:2226 36755125 10.1038/s41598-023-29602-z PMC9906574

[5] Nudelman NT et al (2025) Markedly Increased prevalence of eosinophilic esophagitis in patients with atopic diseases in a US Veteran Population. Int Arch Allergy Immunol 1–8. 10.1159/000547753 10.1159/000547753 40875695

[6] Wojas O et al (2025) The Overlap of Allergic Disorders and Upper Gastrointestinal Symptoms: Beyond Eosinophilic Esophagitis. Nutrients 17:1355 40284218 10.3390/nu17081355 PMC12030484

[7] Maret-Ouda J, Markar SR, Lagergren J (2020) Gastroesophageal Reflux Disease JAMA 324:2536 33351044 10.1001/jama.2020.21573

[8] Bertin L et al (2025) Pathophysiology of GERD. Digestion. 10.1159/000547023 40562014 10.1159/000547023 PMC12279320

[9] Leonardi A, Castegnaro A, Valerio ALG, Lazzarini D (2015) Epidemiology of allergic conjunctivitis: clinical appearance and treatment patterns in a population-based study. Curr Opin Allergy Clin Immunol 15:482–488 26258920 10.1097/ACI.0000000000000204

[10] Ravn NH et al (2021) Bidirectional association between atopic dermatitis, conjunctivitis, and other ocular surface diseases: A systematic review and meta-analysis. J Am Acad Dermatol 85:453–461 33253849 10.1016/j.jaad.2020.11.037

[11] Ludwig RJ et al (2025) A comprehensive review of methodologies and application to use the real-world data and analytics platform TriNetX. Front Pharmacol 16 10.3389/fphar.2025.1516126 PMC11931024 40129946

[12] Bao J, Wen Y, Tian L, Jie Y (2025) Decoding Allergic Conjunctivitis: Latest Perspectives on Etiological Drivers and Immunopathological Mechanisms. Clin Rev Allergy Immunol 68:85 40879847 10.1007/s12016-025-09098-3 PMC12397188

[13] Aziz S et al (2025) Unravelling the pathogenesis of Eosinophilic Esophagitis from genetic predisposition to environmental triggers. Clin Exp Immunol 219 10.1093/cei/uxaf039 PMC12232548 40581073

[14] Capucilli P, Hill DA (2019) Allergic Comorbidity in Eosinophilic Esophagitis: Mechanistic Relevance and Clinical Implications. Clin Rev Allergy Immunol 57:111–127 30903437 10.1007/s12016-019-08733-0 PMC6626558

[15] Khokhar D et al (2022) Eosinophilic esophagitis: Immune mechanisms and therapeutic targets. Clin Experimental Allergy 52:1142–1156 10.1111/cea.14196 PMC9547832 35778876

[16] Borges S, Pereira VA, Chang C, Galor A (2025) Where eye meets body part 2: uniting allergy pathways in ocular and atopic disease – T cells take the lead. Curr Opin Allergy Clin Immunol 25:364–37340747966 10.1097/ACI.0000000000001097

[17] Massironi S et al (2023) Mechanistic Insights into Eosinophilic Esophagitis: Therapies Targeting Pathophysiological Mechanisms. Cells 12:2473 37887317 10.3390/cells12202473 PMC10605530

[18] Komi EA, Rambasek D, T., Bielory L (2018) Clinical implications of mast cell involvement in allergic conjunctivitis. Allergy 73:528–539 29105783 10.1111/all.13334

[19] Kay AB (2001) Allergy and Allergic Diseases. N Engl J Med 344:30–37 11136958 10.1056/NEJM200101043440106

[20] Furuta GT, Katzka DA (2015) Eosinophilic Esophagitis. N Engl J Med 373:1640–1648 26488694 10.1056/NEJMra1502863 PMC4905697

[21] Spergel J, Aceves SS (2018) Allergic components of eosinophilic esophagitis. J Allergy Clin Immunol 142:1–8 29980277 10.1016/j.jaci.2018.05.001 PMC6083871

[22] Capucilli P, Cianferoni A, Grundmeier RW, Spergel JM (2018) Comparison of comorbid diagnoses in children with and without eosinophilic esophagitis in a large population. Ann Allergy Asthma Immunol 121:711–716 30194971 10.1016/j.anai.2018.08.022

[23] González-Cervera J et al (2017) Association between atopic manifestations and eosinophilic esophagitis. Ann Allergy Asthma Immunol 118:582–590e2 28366582 10.1016/j.anai.2017.02.006

[24] Wojas O et al (2025) The Overlap of Allergic Disorders and Upper Gastrointestinal Symptoms: Beyond Eosinophilic Esophagitis. Nutrients 17:1355 40284218 10.3390/nu17081355 PMC12030484

[25] Moawad FJ et al (2015) Comparison of eotaxin-3 biomarker in patients with eosinophilic oesophagitis, proton pump inhibitor-responsive oesophageal eosinophilia and gastro-oesophageal reflux disease. Aliment Pharmacol Ther 42:231–238 26011446 10.1111/apt.13258

[26] Kia L, Hirano I (2015) Distinguishing GERD from eosinophilic oesophagitis: concepts and controversies. Nat Rev Gastroenterol Hepatol 12:379–386 25986303 10.1038/nrgastro.2015.75 PMC4948861

[27] Robson J et al (2019) Sensitivity and Specificity of Administrative Medical Coding for Pediatric Eosinophilic Esophagitis. J Pediatr Gastroenterol Nutr 69 10.1097/MPG.0000000000002340 30921258

[28] Low EE et al (2023) Development and Validation of the Veterans Affairs Eosinophilic Esophagitis Cohort. Clin Gastroenterol Hepatol 21:3030–3040e4 37031716 10.1016/j.cgh.2023.03.033 PMC10558623

